# Examining the Interaction between Exercise, Gut Microbiota, and Neurodegeneration: Future Research Directions

**DOI:** 10.3390/biomedicines11082267

**Published:** 2023-08-14

**Authors:** Daniel Rojas-Valverde, Diego A. Bonilla, Luis M. Gómez-Miranda, Juan J. Calleja-Núñez, Natalia Arias, Ismael Martínez-Guardado

**Affiliations:** 1 Nucleus of Studies for High Performance and Health (CIDISAD-NARS), School of Human Movement Sciences and Quality of Life (CIEMHCAVI), National University, Heredia 86-3000, Costa Rica; 2 Sports Injury Clinic (Rehab & Readapt), School of Human Movement Sciences and Quality of Life (CIEMHCAVI), National University, Heredia 86-3000, Costa Rica; 3 Research Division, Dynamical Business & Science Society—DBSS International SAS, Bogotá 110311, Colombia; dabonilla@dbss.pro; 4 Research Group in Biochemistry and Molecular Biology, Faculty of Sciences and Education, Universidad Distrital Francisco José de Caldas, Bogotá 110311, Colombia; 5 Research Group in Physical Activity, Sports and Health Sciences (GICAFS), Universidad de Córdoba, Montería 230002, Colombia; 6 Sport Genomics Research Group, Department of Genetics, Physical Anthropology and Animal Physiology, Faculty of Science and Technology, University of the Basque Country (UPV/EHU), 48940 Leioa, Spain; 7 Sports Faculty, Autonomous University of Baja California, Tijuana 22615, Mexico; luismariouabc@gmail.com (L.M.G.-M.); juan.calleja@uabc.edu.mx (J.J.C.-N.); 8 BRABE Group, Department of Psychology, Faculty of Life and Natural Sciences, University of Nebrija, C/del Hostal, 28248 Madrid, Spain; narias@nebrija.es

**Keywords:** microbial diversity, metabolic function, neurodegenerative disease, inflammation, exercise interventions, gut–brain axis, lactate, immune system

## Abstract

Physical activity has been demonstrated to have a significant impact on gut microbial diversity and function. Emerging research has revealed certain aspects of the complex interactions between the gut, exercise, microbiota, and neurodegenerative diseases, suggesting that changes in gut microbial diversity and metabolic function may have an impact on the onset and progression of neurological conditions. This study aimed to review the current literature from several databases until 1 June 2023 (PubMed/MEDLINE, Web of Science, and Google Scholar) on the interplay between the gut, physical exercise, microbiota, and neurodegeneration. We summarized the roles of exercise and gut microbiota on neurodegeneration and identified the ways in which these are all connected. The gut–brain axis is a complex and multifaceted network that has gained considerable attention in recent years. Research indicates that gut microbiota plays vital roles in metabolic shifts during physiological or pathophysiological conditions in neurodegenerative diseases; therefore, they are closely related to maintaining overall health and well-being. Similarly, exercise has shown positive effects on brain health and cognitive function, which may reduce/delay the onset of severe neurological disorders. Exercise has been associated with various neurochemical changes, including alterations in cortisol levels, increased production of endorphins, endocannabinoids like anandamide, as well as higher levels of serotonin and dopamine. These changes have been linked to mood improvements, enhanced sleep quality, better motor control, and cognitive enhancements resulting from exercise-induced effects. However, further clinical research is necessary to evaluate changes in bacteria taxa along with age- and sex-based differences.

## 1. Introduction

Exercise has long been recognized as an important strategy for maintaining overall health and improving well-being. In recent years, scientists have begun to understand the complex relationship between exercise and microbiota [1,2]. The gut microbiota, popularly known as gut flora, refer to the trillions of microorganisms that reside in the gastrointestinal tract, especially bacteria, which are the most abundant and most studied. These microorganisms play a critical role in maintaining optimal function of the gut as well as the overall body health.

Experimental evidence has shown that the gut microbiota can be modified by a variety of factors, including diet, physiological stress, and antibiotic use [3,4]. Importantly, physical exercise has been recognized as an important modulator of the gut microbiota. Indeed, studies have shown that regular exercise is associated with a more diverse and stable gut microbiota, which is associated with better gut health [5,6,7]. For example, cardiovascular exercise (e.g., running or cycling) has been found to increase the abundance of certain bacterial species, such as *Akkermansia muciniphila*, *Faecalibacterium prausnitzii*, *Prevotella*, *Methanobrevibacter*, and *Veillonella atypica* [8,9], as part of the exercise-induced physiological adaptation processes. Besides increasing the abundance of beneficial bacteria, exercise has also been found to have anti-inflammatory effects on the gut by decreasing the levels of pro-inflammatory cytokines in the body while promoting immunosurveillance [10]. It seems that exercise has a positive effect on gut permeability, avoiding the “leaky gut” [11]. A leaky gut is characterized by a porous gut lining, which allows harmful substances and bacteria to leak into the bloodstream, leading to inflammation [12]. Regular exercise has been found to help strengthen the gut barrier, reducing the risk of developing a leaky gut [13]. It is worth noting that inflammation in the gut has been linked to a variety of conditions, including irritable bowel syndrome, inflammatory bowel disease, and even mental health disorders [14].

The relationship between exercise and gut health is complex, and more research is needed to fully understand the effects and mechanisms involved. Some studies suggest that high-intensity exercise may have a negative impact on the gut, while others have shown no significant difference between high- and low-intensity exercise interventions [15]. Certainly, the magnitude of the exercise-induced stress is key to evaluating its effects on human physiology [16], including changes in gut microbiota. For instance, it has been discussed that excessive exercise and inadequate recovery not only strongly affect the gastrointestinal system negatively [17] but also impair gut microbiota composition and function [18]. This negative effect normally leads to a dysbiosis that may contribute, at least in part, to worsened immune responses that are seen during overtraining [18]. Moreover, it is also important to consider other factors such as diet (e.g., fluid restrictions), sleep, environmental conditions (e.g., altitude, temperature), trainability, age, and stress levels, as they also impact gut health [19]. Thus, psychological stress and exercise-induced stress (i.e., intensity and/or duration of the exercise stimuli) affect microbiota [20].

Scientific evidence has highlighted the intricate interactions between gut health, gut microbiota, and neurodegenerative diseases, suggesting that changes in gut microbial diversity and function might have an important role in the onset and progression of these neurological conditions [21,22]. Additionally, recent studies have revealed a dynamic interplay between gut microbiota, neurodegeneration, and the role of physical activity [23,24]. Regular physical exercise has been shown to have a positive effect on gut health, specifically on gut microbiota, by increasing the abundance of beneficial bacteria, reducing gut inflammation, and improving gut barrier function. However, the relationship between these factors is complex and multifactorial; therefore, it is not fully understood. How do these interactions vary due to different factors such as population, type of exercise, and others? What are the specific mechanisms by which gut microbiota, neurodegeneration, and physical activity are linked? This article aims to review the current literature on the interplay between exercise, gut microbiota, and neurodegeneration. We will emphasize the convergence of the physiological pathways by which physical exercise impacts the gut microbiome and the brain.

## 2. Methods

This study follows previous guidelines on the development of a narrative review outlined by Dixon-Woods et al. [25] and Popay et al. [26]. It encompasses the identification, selection, evaluation, and synthesis of the published articles. The first author organized and recruited experts on different areas regarding the aim of the narrative review. The authors collaborated remotely to establish the goals and objectives of the review through a series of online meetings and email correspondence. Each author then contributed a section that aligned with their individual expertise (e.g., nutrition, sport science, aging), resulting in the creation of a first draft of the manuscript. This draft was subsequently reviewed and discussed among all authors following previously an established methodology [27], with several rounds of revisions and refinements made before the final approval. All communications and coordination throughout the process was completed electronically and was led by the first author.

### 2.1. Eligibility Criteria

All relevant types of articles were considered, including meta-analyses, systematic reviews, randomized controlled trials (RCTs), exploratory studies, confirmatory studies, and case reports. Preference was given to high-quality research, such as meta-analyses and RCTs. There were no date restrictions.

### 2.2. Information Sources

The primary sources for the articles included the following online databases: PubMed/MEDLINE, Web of Science, and Google Scholar. The studies were published between 2013 and 2023.

### 2.3. Search Strategy

The search string included free terms as “neuromodulation”, “gut health”, “exercise”, “neurodegeneration”, “neurodegenerative diseases”, and “microbiota”. Each term was combined with keywords such as long-term, chronic, acute, psychiatry, pathophysiology, injury, illness, and disease. The reference lists of the selected articles were also manually searched for additional literature (snowballing).

### 2.4. Findings Presentation

The narrative discussion by each author was aligned with the author’s individual expertise (e.g., nutrition, sport science, aging) and interpretation of the relevant articles. The text provides details on the nature of each study organized by sections including: (i) physical exercise, microbiota, and health; (ii) the neuromodulatory effects of physical exercise; (iii) exercise and neurodegeneration; and (iv) a discussion of potential convergent hallmarks in the complex interaction between physical exercise, microbiota, and neurodegeneration. Finally, future directions are presented to guide upcoming research in the field.

## 3. Physical Exercise–Gut Health Relationship

Stress can be defined as the perturbance of any biological system by modifying its components after external (e.g., exercise or diet intervention) or internal (e.g., genetics, prior knowledge, and current adaptations) stimuli. According to the allostasis–interoception model [28], to evoke a healthy biological adaptation in the individual, stress should be maintained in a chronic and periodized manner, while the system must pay the cost for it (i.e., allostatic load). If the magnitude of stress overcomes the system’s capacity, an allostatic overload arises, and a pathological state might take place [29]. This has been demonstrated to occur at the physiological and cellular level (for detailed information see the following reviews and meta-analysis: [30,31,32]). Along this line, stress and allostatic load are believed to be significant factors in the relationship between sex/gender and cardiovascular diseases. Longpré-Poirier et al. [33] posit that chronic stress and psychosocial factors may better account for the patterns of increased allostatic load observed in women. On the other hand, biological risk factors and unhealthy behaviors may play a more crucial role in driving increased allostatic load in men. Notably, men exhibit allostatic load patterns that are closely linked to impaired anthropometric, metabolic, and cardiovascular functioning, while women tend to have greater dysregulation in neuroendocrine and immune functioning. Additionally, Wang et al. [34] utilized an integrated micromechanical tool capable of applying controlled mechanical stress to individual cells and simultaneously monitored dynamic subcellular mechanics, observing a biphasic process in individual cell allostasis. This process involves cellular mechanics attempting to return to a stable state through a mechanoadaptive phase with heightened biophysical activity, followed by a decaying adaptive phase. The observations suggest that cellular allostasis is achieved through a complex balance of subcellular energy and cellular mechanics. When subjected to a transient and localized physical stimulation, cells trigger an allostatic state that maximizes energy and surmounts a mechanical “energy barrier”, followed by a relaxation state that achieves mechanobiological stabilization and minimizes energy expenditure.

Exercise-induced stress has long been recognized for its numerous benefits to physical and mental health [35]. It has been shown that an effective exercise dose might increase the production of anti-inflammatory cytokines (e.g., interleukin-10) while at the same time decreasing pro-inflammatory molecules (e.g., interleukin-6) [36]. This can help to reduce the overall level of inflammation in the body, which might result in an enhanced immune response (i.e., immunosurveillance) [10]. However, recent research has highlighted the interplay between exercise, inflammation, and gut health as a plausible mechanism for the immunomodulatory effects that are connected to exerkine production [35]. Exerkines are molecules that are characterized as signaling agents and released in response to both acute and chronic exercise. These molecules exert their effects through various pathways, including endocrine, paracrine, and autocrine mechanisms. Numerous organs, cells, and tissues release these factors, with examples including skeletal muscle releasing myokines, the heart releasing cardiokines, the liver releasing hepatokines, white adipose tissue releasing adipokines, brown adipose tissue releasing baptokines, and neurons releasing neurokines. The potential roles of exerkines are vast and encompass improvements in cardiovascular health, metabolic function, immune response, and neurological well-being [37,38,39,40]. This suggests that regular physical activity might have a positive impact on the gut microbiota (diversity and function) [1,2] which may also facilitate healthy metabolic shifting [41].

The gut microbiota refers to communities of microorganisms that are made up of mainly Bacteria, Archaea, and Eukarya (fungi, protozoans, and metazoan parasites), as well as eukaryotic and prokaryotic viruses (bacteriophages) that reside in the gastrointestinal tract [42]. As any other human biological component that contribute to the physiological regulation, dysbiosis, or an imbalance in the gut microbiota, has been linked to a variety of health issues such as inflammatory disorders [43]. Since inflammation is a key aspect of many chronic diseases (e.g., obesity, diabetes), the gut microbiota has been described to play a crucial role in disease prevention and management through the production of short-chain fatty acids (SCFAs), anti-inflammatory molecules, and subsequent modulation of the immune response [44].

Notably, physically active individuals have a higher abundance of beneficial bacteria and a lower abundance of pro-inflammatory bacterial species [18,44]. In this regard, exercise favors the production of SCFAs by gut microbiota, which can also improve gut-barrier function [45,46]. These exercise-mediated effects on gut health are not limited to healthy individuals, given that regular physical activity can also improve the gut status in individuals with chronic diseases [25]. In the obese population, regular physical activity has been shown to improve gut microbial diversity, which subsequently contributes to a reduction in systemic inflammation [47]. In general, regular exercise has been shown to improve disease symptoms (e.g., abdominal pain) and reduce the need for medication.

Notwithstanding, how does physical exercise regulate the gut microbiota status? Based on the current research, we might establish that it is mediated by exerkines, especially lactate (La^−^). It is worth noting that La^−^ is a stress-related signaling molecule that plays a key role in allodynamic responses in health and disease [48]. Therefore, it is a biomarker that is frequently used in exercise and sport physiology, as it positively correlates with intensity (stress level). La^−^ is an intermediate product of energy metabolism that is considered one of the key stress-related molecules of human physiology in health and disease rather than a waste or fatigue substance [49]. Some of the pleitropic effects of La^−^ metabolism include: (i) regulation of energy production (e.g., Cori’s cycle, transient between glycolysis and oxidative metabolism, changes in substrate utilization); (ii) organelle signaling and interoception processes (i.e., cross-talking between organelles and tissues via monocarboxylate transporter isoforms (MCTs)); and (iii) epigenetic control of gene expression (lactylation) [50]. Indeed, exercise training has been shown to enhance the expression of MCT1 and MCT4, which contributes to the higher transport and removal rate of La^−^ [51].

Interestingly, La^−^ disposal, production, and transportation are not only regulated by extrinsic factors such as exercise dose and energy intake (i.e., distal physiology) but also by intrinsic factors like genetic variations in La^−^-related genes (MCTs) and the microbiota status [52,53]. In recent years, this direct interaction between La^−^ levels and the microbiota status has been reported in different phenotypes, including the obese population and highly trained athletes [54,55,56]. *Veillonella atypica*, *Eubacterium hallii group*, *Anaerobutyricum hallii*, *Anaerostipes*, and many other bacterial species can metabolize La^−^ to produce SCFAs and other intermediates that contribute to the microbial diversity and to the enrichment of specific bacterial populations after an exercise period [45]. It should be noted that MCT1 is present as a myocyte membrane transporter and is also expressed in the gut epithelium to facilitate the absorption of SCFAs produced by the gut microbiota [57]. Alternatively, it is plausible that other La^−^ sources beyond the muscle (e.g., bacterial species such as *Lactobacillus* spp.) impact exercise-induced adaptations by increasing the La^−^ availability to allow La^−^-utilizing bacteria to produce butyrate and other SCFAs [53]. It seems that this bidirectional interaction mediated by changes in La^−^ levels may be responsible, at least in part, for the exercise-induced changes in the microbiota and the bacteria-related contribution to energy metabolism (SCFAs) and exercise adaptations at the physiological level. Nevertheless, further research is warranted to examine the minimal exercise intensity and the necessary time of an exercise training program required to positively alter the microbiota status.

## 4. Physical Exercise as a Neuromodulator

Physical exercise, regardless of the intensity level, has been proven to be an effective treatment for a wide range of medical conditions. These include cardiovascular [58,59], respiratory [60,61], metabolic [62,63,64], musculoskeletal [65,66], and neurological [67,68,69] conditions. Research suggests that exercise and physical activity can lead to changes in brain function and improve the ability to adapt to new challenges and behavior changes [70,71,72]. Additionally, several studies have highlighted the significance of cortisol in certain neurological conditions. The conversion of cortisol to cortisone, in fact, has been shown to increase proportionately with exercise as a response to training. This is essential, as it protects individuals who have undergone training from the negative effects of prolonged elevated cortisol levels [73], including depressive issues and anorexia [74]. The exercise, sport science, and medicine community should delve deeper into the connection between exercise and neural function to further understand the neurobiological mechanisms active during various types of physical activity.

Endurance training in various forms and intensities has been shown to increase endorphins and endocannabinoids, resulting in reduced symptoms of anxiety, sleep disorders, and depression [75,76]. Anandamide, a type of endocannabinoid that is increased during exercise, has been linked to the regulation of physical and psychological stress [69]. In this regard, anandamide might play a role in various brain activities through physiological regulation of stress, anxiety, and post-stress recovery [77]. This can lead to a reduction in overactivity in the amygdala [78]. It should be noted that regulation of the stress response after physical exercise is dependent on the glucocorticoid hormone. Since anandamide is a fatty acid-like molecule, it can readily pass through the blood–brain barrier and contribute to mood regulation via the glucocorticoid pathway [79]. Moreover, there is strong evidence to suggest that anandamide might have a significant role in the increase in brain-derived neurotrophic factor (BDNF) during and after exercise. In fact, anandamide levels remain elevated during recovery, delaying the return to normal levels of BDNF [80]. BDNF is considered the primary molecule responsible for exercise-induced neurogenesis and brain plasticity, in addition to its beneficial effects on learning through its enhancement of synaptic plasticity and long-term potentiation [81].

**Figure 1:**
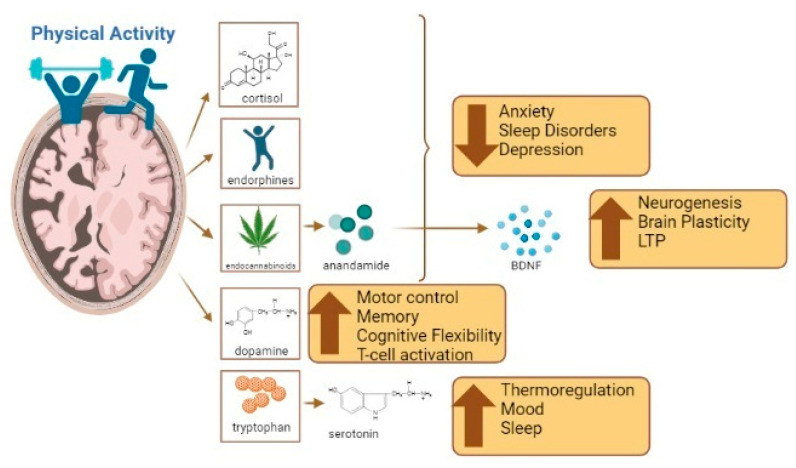
The benefits of exercise and its influence on brain functions such as anxiety, sleep, mood regulation, cognition, and inflammation. Created using Biorender.com (accessed on 4 August 2023).

Furthermore, exercise increases the likelihood of tryptophan crossing the blood–brain barrier, increasing serotonin levels in the brain. This is due to an increase in the uptake of branched-chain amino acids in muscles during exercise [82]. Serotonin is a neurotransmitter that affects thermoregulation, mood, emotional behavior, food intake, and sleep–wake cycles [83]. However, excessive serotonin levels can lead to neurological issues, including mental and autonomic disorders [84]. Dopamine, another neurotransmitter that is increased during and after exercise, plays a role in the early stages of motor control, memory, and cognitive flexibility [85]. Dysfunction in dopamine levels can lead to various conditions, such as schizophrenia, attention deficit hyperactivity disorder, bipolar depression, addiction, and Parkinson’s disease [86]. Dopamine also regulates immune functions related to T-cell activation and inflammation. [87]. Its receptors play an important role in synaptic plasticity and motor behavior by reinforcing the selection of movements. Considering the aforementioned points, both short- and long-term exercise programs have been shown to enhance cognitive performance and delay neurodegenerative responses. Exercise appears to modulate levels of neurotrophins (e.g., BDNF), hormones (e.g., cortisol), and neurotransmitters (including anandamide, dopamine, and serotonin); however, these effects vary based on factors such as sex, age, and genetics [79]. This neuroregulation caused by exercise appears to be dependent on the intensity of the exercise [88,89] (see Figure 1).

## 5. The Role of Exercise in Neurodegeneration

There is robust evidence showing that exercise can enhance neurological function in both healthy adults and those with cognitive impairments [90]. Research suggests that cardiovascular exercise, in particular, can enhance cognitive abilities such as processing speed, attention, and cognitive flexibility [68]. Similarly, strength exercise can improve physical capabilities as well as mental and behavioral conditions [90]. Physical exercise provides certain benefits and affects gut health, brain function, and cognitive function through different pathways. In the following paragraphs, we briefly describe common neurodegenerative diseases and the potential for exercise to alleviate symptoms as part of a non-pharmacological strategy.

Parkinson’s disease is a prevalent neurodegenerative disorder that causes progressive and unpredictable damage to the brain [91]. Characterized by the death of dopamine-producing neurons in the brain, Parkinson’s disease is characterized by motor dysfunctions, such as difficulty initiating and performing voluntary movements, issues with posture, stiffness, slow movement, muscle rigidity, and problems with coordinating movement sequences. It also often results in behavioral and cognitive impairments [92,93,94]. Exercise is often recommended as a strategy to manage the symptoms and disability caused by Parkinson’s disease. Exercise-based programs such as hydrotherapy have been shown to be effective in treating some symptoms of Parkinson’s disease, including improved motor function, balance, and quality of life [94,95,96]. Alternative therapies like Tai Chi [97], yoga [98], and dance [99] may also help treat Parkinson’s disease and improve outcomes like gait, balance, and functional mobility. Other programs like Nordic walking [100], resistance training, and flexibility training have also been effective in improving motor symptoms and functional performance in Parkinson’s disease patients. Strength programs usually accompanied by stretching, balance, and breathing exercises also suggest improvements in physical and cognitive capabilities [101].

Alzheimer’s disease is a progressive and degenerative disorder that affects memory and cognitive function. It is the primary cause of dementia among adults, with age being the main risk factor [102]. Exercise has been shown to be an effective alternative and complementary approach to medication in Alzheimer’s due to it having fewer side effects and better adherence compared to drugs [103]. Cardiovascular exercise can reduce the prevalence, morbidity, and mortality caused by Alzheimer’s and slow down the rate of decline [102]. Exercise has a neuroprotective role, promoting greater angiogenesis and neurogenesis, reducing inflammation, and decreasing cerebrovascular risk factors [104,105,106]. Long-term exercise programs can prevent the risk factors of Alzheimer’s disease, improve blood flow, increase hippocampal volume, and improve neurogenesis [103]. A variety of activities, such as swimming, walking, cycling, yoga, and bowling, have been shown to improve cognitive performance, memory, and executive function. Moreover, a resistance exercise-based program of one hour per week can help reduce the progression of Alzheimer’s by improving strength, flexibility, and balance in the long-term [102,107]. Studies also suggest that exercise can preserve the volume and integrity of the hippocampus, temporal, basal ganglia, and thalamus [108].

Multiple sclerosis (MS) is a chronic disorder of the central nervous system in which the patient’s immune system attacks the myelin sheath surrounding the axons of neurons in the brain and spinal cord [109]. This leads to demyelination, which causes symptoms such as a loss of function and feeling in the limbs, chronic pain, fatigue, balance loss, and cognitive impairment [110]. There is currently no cure for MS, and evidence suggests that MS patients are less active than the general population [109]. Various exercise modalities, such as cardiovascular, strength, and interval training, have been used to treat MS. These interventions, such as cycling and walking–jogging, can help mitigate declines in walking mobility and reduce disease progression [111]. A systematic review found that cardiovascular and mixed exercise can reduce self-reported fatigue in MS patients [112].

Finally, amyotrophic lateral sclerosis (ALS) is a progressive, fatal, and neurodegenerative disease characterized by symptoms such as fatigue, muscle stiffness, and cognitive impairment [113]. The role of exercise in the treatment of ALS is controversial, but when implemented early in the disease, it can help improve motor function and enhance independence [114,115]. Rehabilitation programs usually focus on avoiding muscle fatigue and damage, and the exercises used include swimming, walking, and cycling at submaximal levels [116].

## 6. Complex Interactions between Exercise, Neurodegeneration, and Gut Health

The diversity and composition of the gut microbiota are crucial for several vital functions, including regulation of basic processes such as digestion, as well as facilitating the extraction, synthesis, and absorption of nutrients and metabolites [117]. Furthermore, the gut microbiota status determines the abundance of metabolites, neurotransmitters, and SCFAs produced by the bacteria [118].

In recent years, interest in the connection between the gut microbiota and the gut–brain axis has raised significantly, particularly in relation to neurodegenerative disorders. This is due to evidence suggesting that gut microbiota imbalances might play a role in pathological processes associated with psychiatric and neurological conditions [119]. It has been previously described that the gut plays a crucial role in releasing various hormones, peptides, and microbial metabolites, such as SCFAs, secondary bile acids, and products derived from tryptophan and polyphenols. These substances have significant effects on neuronal function and survival. Notably, many of these compounds can cross the blood–brain barrier (BBB), including SCFAs, which exploit active membrane transporters on the endothelium to reach the central nervous system (CNS) [120]. Conversely, the CNS also sends efferent responses to the gut, thereby regulating important aspects like motility, mucus secretion, barrier integrity, and visceral sensitivity [121]. The communication between the gut and the CNS is bidirectional, and this is why any dysbiosis in the microbiota would impact brain function through this gut–brain axis.

Dysregulation of the gut microbiota has been linked to various neurodegenerative disorders such as Parkinson’s, Huntington’s, multiple sclerosis, and Alzheimer’s [122,123,124]. These diseases have been associated with a decline in the integrity and function of the gut, potentially resulting in increased gut permeability and inflammation. This can create an abnormal environment in the gut [119], which disrupts communication between the gut and the brain. Communication between the gut microbiota and the nervous system may be driven by gut–brain axis pathways that include the enteric nervous system, vagus nerve neuronal connections, the immune system, and metabolism [125]. The enteric nervous system is composed of enteroendocrine cells which receive signals directly form the gut microbiota. These cells can induce the secretion of hormones that cross the BBB and impact the function of brain cells. Furthermore, the vagus nerve is intricately connected to enteroendocrine cells, and it serves as a potentially crucial link between the gut microbiota and the brain. This direct connection allows for bidirectional communication between the gut and the brain, enabling the exchange of signals and information that can influence various physiological and neurological processes. The vagus nerve’s involvement in this communication pathway highlights its importance in mediating the gut–brain axis, which could be through exercise and could facilitate interactions between the gut microbiota and brain function. Additionally, immune-signaling mediators such as cytokines, chemokines, and microbial-associated molecular patterns (MAMPs) play a crucial role in facilitating communication between the gut microbiota and the brain. These mediators can interact through both direct and indirect pathways, enabling bidirectional signaling between the gut and the brain. Through these signaling pathways, the gut microbiota can influence immune responses and neuroinflammation in the brain, while the brain can also modulate immune functions in the gut. This intricate immune communication network contributes to the complex interactions of the gut–brain axis and plays a significant role in shaping overall health and well-being. Finally, it is also important to note that products of microbial metabolism, such as short-chain fatty acids (SCFAs) and other microbial-derived metabolites such as tryptophan, act as chemical signals in the host’s cells, influencing various aspects of cellular function [126].

Moreover, clinical research has associated gut microbiota imbalances with neurodegenerative disorders [22]. Exercise, therefore, can improve gut health by increasing the diversity of the microbiota and promoting a balance between the beneficial and harmful bacterial communities [127,128]. This suggest that a positive impact on the gut microbiota might influence neurological health [129,130]. Exercise can decrease the transit time of food through the gastrointestinal tract, reducing the exposure of pathogens to the mucus layer in the gut and having a secondary effect on the circulatory system, which in turn reduces the population of harmful pathogens [119,131].

The communication between the gut and the central nervous system (CNS) is very complex, with microbial metabolites such as SCFAs, bile acids (BAs), and tryptophan playing a key role. These compounds bind to receptors in the CNS and affect various functions, including intestinal transport, secretion, and permeability. Additionally, signals from the gut are sent to the CNS through the vagus nerve and other channels, influencing feeding behavior and energy homeostasis. Skeletal muscle also plays a role in this communication, with receptors for SCFAs and BAs found on muscle fibers. This allows the gut microbiota to participate in muscle energy metabolism and fiber conversion. Furthermore, during exercise, myokines secreted by skeletal muscle stimulate the secretion of intestinal hormones, which can further influence food intake and energy balance. The concept of the brain–gut–muscle axis is becoming increasingly recognized as important for regulating energy homeostasis and overall health [132].

The mechanisms by which exercise affects the gut microbiome and alters its components have been studied. A strong connection between exercise, stress-related factors, and the immune response is thought to be the key mediating pathway [119,133]. Animal studies (i.e., mice and rats) have demonstrated that exercise leads to an increase in antioxidant enzymes, anti-inflammatory cytokines, and proteins that prevent cell death in intestinal lymphocytes while also decreasing proinflammatory cytokines and proteins that promote cell death. This leads to a reduction in intestinal inflammation [134,135] and immunosurveillance [10], which has been reported in clinical research (see Table 1).

**Figure 2:**
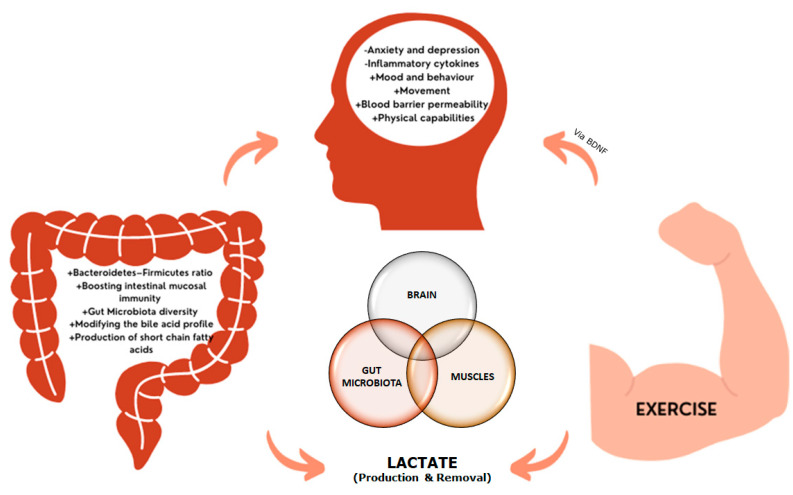
The brain-gut-muscle axis. Increases in lactate, characteristic of neurodegenerative diseases, are controlled by exercise, which regulates systemic and brain lactate levels. Additionally, changes in the microbiota diversity and intestinal profile affect to the production of SFCAs, which can cross the blood brain barrier. Under neurodegenerative conditions, changes in mood, behavior, and cognition, together with alterations in the blood-brain barrier and the inflammatory state, have been reported, leading to neuronal death. The release of brain-derived neurotrophic factor (BDNF) during and after exercise contributes to the neuroplasticity, improving the neurodegenerative condition.

From a molecular point of view, it is necessary to highlight that La^−^ metabolism is at the convergence between exercise, microbiota, and neurobiology. We have already discussed the influence of exercise-induced stress on microbiota status via higher La^−^ levels and increased MCTs content, as well as the enrichment of La^−^-utilizing bacterial species in the gut and the subsequent higher production of SCFAs to mediate exercise adaptations. However, abnormal elevated and sustained La^−^ concentrations have been linked to the progression of major cellular pathologies that are associated with neurodegenerative diseases [136]. While physical exercise enhances the flux of SCFAs and La^−^ through an increased expression of MCTs, in the progression of neuropathological diseases, the tissues are not able to participate in the sequestration and utilization of La^−^, resulting in an allostatic overload [137]. A recent meta-analysis on post-mortem and in vivo imaging data concluded that increased La^−^ levels and reduced pH are common features of the schizophrenic brain [138]. In addition, there is a marked association between La^−^ concentrations, hyperphosphorylation of Tau (τ) proteins, and cognitive decline in Alzheimer’s disease [139]. In general, La^−^ is a stress-related signaling molecule, and the La^−^ production/removal ratio can be positively modified by physical exercise (i.e., higher MCT expression, changes in lactate threshold, and metabolic shifting) [140]. Thus, it is plausible to state that exercise and microbiota regulate the systemic and brain levels of La^−^ through a feedforward positive motif that might result in energy optimization and control of oxidative stress and hydrogen ion (H^+^) concentrations. These key features are the core for controlling inflammation and possibly contribute to the preventive and treatment of neurological disorders. This relationship between exercise, gut microbiota health, and cognitive function (gut–brain–muscle axis) is shown in Figure 2.

Given the link between gut dysfunction and the gut microbiome in neurodegenerative diseases and the effects of physical exercise on the gut microbiome, further research is needed to confirm whether exercise can partially modulate neurodegeneration through the gut microbiome [119,141]. One proposed mechanism is linked to the improvement in mitochondrial dysfunction found in neurodegenerative disorders. It has been shown that both acute and chronic exercise can initiate dynamic processes in mitochondria, including biogenesis, fusion, fission, and mitophagy [142]. One study found that exercise training can enhance the metabolic and genetic capabilities associated with the tricarboxylic acid (TCA) cycle. In contrast, non-exercised mice with obesity induced by a high-fat diet (HFD) exhibited a reduced metabolic capacity in their fecal microbiota [143]. These findings suggest that exercise has a positive impact on mitochondrial function and the gut microbiota, potentially contributing to improved neurodegeneration. The implementation of multimodal strategies that include exercise, diet, sleep hygiene, and psychological therapy has been shown to be highly relevant in the treatment and management of patients with degenerative diseases. By addressing both physical and mental health needs, multimodal treatments have the potential to slow the progression of degenerative diseases, improving the quality of life and overall well-being of patients. It is important for healthcare professionals to consider a holistic approach for treating these conditions to achieve optimal results.

## 7. Future Research Directions

Research on the interplay between gut microbiota, neurodegeneration, and physical activity is an emerging field that is rapidly gaining attention in the scientific community. Future research in this area will likely focus on several key areas.

First, more research is needed to understand the specific mechanisms by which gut microbiota, neurodegeneration, and physical activity are linked. For example, studies are needed to confirm the convergence of La^−^ metabolism and also to identify the specific gut microbial species and alternative metabolic pathways that may play a role in neurodegeneration, as well as the precise ways in which exercise impacts the gut microbiome.

Second, further clinical research is warranted to understand the acute and chronic responses among different populations. It is important to understand how these interactions vary among different age groups, sexes, ethnicities, and lifestyles, as these factors may play a role in the susceptibility to neurodegeneration.

Third, the field will likely move towards an integrated perspective to study gut microbiota, neurodegeneration, and physical activity by considering the impact of both diet and stress on the gut–brain axis. Diet is known to play a crucial role in shaping the gut microbial ecosystem. Since stress can exacerbate inflammation in the gut, it is necessary to consider effective dietary interventions (e.g., probiotic and prebiotic intake) in conjunction with an exercise program, which may improve gut microbial health and prevent or slow down the progression of neurodegeneration [144].

Lastly, it will be important to consider the ethical implications of manipulating the gut microbiome to treat or prevent neurodegenerative diseases. Researchers will need to consider the potential risks and benefits of such interventions as well as the potential long-term effects on gut microbial health and overall well-being.

## 8. Conclusions

Studies have shown that the gut microbiota plays a crucial role in maintaining overall health and well-being, and that there is a dynamic interplay between physical exercise, the gut microbiota, and neurodegeneration. Regular and effective exercise has been shown to modulate gut microbial diversity and function, with positive implications for gut health and overall well-being.

Although more research is needed, it seems that La^−^ metabolism is the convergent mechanism by which physical activity, the gut microbiota, and neurodegeneration progression are linked. Several studies have shown the effects of exercise on the La^−^ production/removal ratio and La^−^ flux regulation, the La^−^-consuming function of certain bacterial species (e.g., *Veillonella atypica*, *Eubacterium hallii group*, *Anaerobutyricum hallii*, and *Anaerostipes*, among others), and the pathophysiological concentrations of La^−^ associated with neurodegenerative disease progression. More research is needed to discover the time course and features of these complex interactions. It is also important to consider the ethical implications of manipulating the gut microbiome to treat or prevent neurodegenerative diseases. Researchers should consider the potential risks and benefits of such interventions, as well as the potential long-term effects on gut microbial health and overall well-being. Future research should focus on developing and testing interventions to improve gut microbial health and prevent or slow down the progression of neurodegenerative diseases.

Overall, the interplay between physical exercise, the gut microbiota, and neurodegeneration is a complex and multifaceted topic that requires further research to fully understand the underlying mechanisms and interactions.

## Figures and Tables

**Table 1:** Description of the positive effects of exercise on gut microbiota and brain functions.

Gut Microbiota Changes	Brain Changes
- Increases *Firmicutes* and *Actinobacteria*	- Decreases anxiety and depression
- Increases butyrate-producing bacteria, such as *Roseburia hominis*, *Faecalibacterium pausnitzii*, and *Ruminococcaceae*	- Improves mood
- Increases butyrate concentration	- Improves motor control
- Reduces transient stool time in the gastrointestinal tract	- Decreases inflammation through t-cell activation
- Increases key antioxidant enzymes (catalase and glutathione peroxidase), anti-inflammatory cytokines (including IL-10), and antiapoptotic proteins (including Bcl-2) in intestinal lymphocytes	- Improves memory, long-term potentiation and cognitive flexibility
- Decreases proinflammatory cytokines (TNF-α and IL-17) and proapoptotic proteins (caspase 3 and 7), leading to an overall reduction in gut inflammation	- Improves sleep
- Increases SCFAs, followed by a decrease in *Bacterioides* and an increase in *Faecalibacterium* and *Lachnospira*	- Improves neurogenesis and brain plasticity
- Modulates gastrointestinal motility	- Improves brain metabolism through mitochondria
